# Metallic Artifact Reduction in Midfacial CT Scans Using Patient-Specific Polymer Implants Enhances Image Quality

**DOI:** 10.3390/jpm13020236

**Published:** 2023-01-28

**Authors:** Julian Lommen, Lara Schorn, Christoph Sproll, Valentin Kerkfeld, Adem Aksu, Frank Reinauer, Norbert R. Kübler, Wilfried Budach, Majeed Rana, Bálint Tamaskovics

**Affiliations:** 1 Department of Oral, Maxillofacial and Facial Plastic Surgery, University Hospital Düsseldorf, Moorenstraße 5, 40225 Düsseldorf, Germany; 2 Karl Leibinger Medizintechnik GmbH & Co. KG, Kolbinger Str. 10, 78570 Mühlheim, Germany; 3 Department of Radiation Oncology, University Hospital Düsseldorf, Moorenstraße 5, 40225 Düsseldorf, Germany

**Keywords:** polyetheretherketone (PEEK), polyetherketoneketone (PEKK), polyphenylsulfone (PPSU), CT artifacts, midface

## Abstract

Midfacial reconstruction after tumor resection surgery is commonly conducted by using autologous bone grafts or alloplastic implants. Titanium is the most frequently used osteosynthesis material in these cases but causes disturbing metallic artifacts in CT imaging. The purpose of this experimental study was to evaluate whether the use of midfacial polymer implants reduces metallic artifacts in CT imaging to improve image quality. Zygomatic titanium (n = 1) and polymer (n = 12) implants were successively implanted in a human skull specimen. Implants were analyzed for their effect on Hounsfield Unit values (streak artifacts) and virtual growth in CT images (blooming artifacts) as well as image quality. Multi-factorial ANOVA and Bonferroni’s post hoc test were used. Titanium (173.7 HU; SD ± 5.1) and hydroxyapatite containing polymers (155.3 HU; SD ± 5.9) were associated with significantly more streak artifacts compared to all other polymer materials. There was no significant difference in blooming artifacts between materials. The metallic artifact reduction algorithm showed no significant difference. Image quality was slightly better for polymer implants compared to titanium. Personalized polymer implants for midfacial reconstruction significantly reduce metallic artifacts in CT imaging which improves image quality. Hence, postoperative radiation therapy planning and radiological tumor aftercare around the implants are facilitated.

## 1. Introduction

Facial reconstruction after tumor resection surgery of oral squamous cell carcinoma (OSCC) is a challenging procedure that aims at restoring oral function and esthetics to lower patient morbidity [1]. In the majority of surgical OSCC therapies, tumor resection includes parts of the maxillary or mandibular bone which often needs to be reconstructed by means of stable osteosynthesis with or without a bone graft [2]. To speed up rehabilitation, tumor resection and concomitant reconstruction are routinely conducted simultaneously in one operation, especially in cases where adjuvant radiation therapy is required [3]. This ensures the postoperative start of radiation therapy as soon as possible to maximize the therapeutic effect. Contemporary osteosynthesis materials for the mandible and midface are made of titanium which provides excellent biocompatibility and mechanical stability to withstand bite forces. It is generally agreed upon that navigation-assisted surgery and patient-specific osteosynthesis are the gold standard for complex reconstructive procedures to provide superior fitting accuracy compared to manually bending plates [4,5]. However, titanium is well known for causing substantial metallic artifacts in computer tomography (CT) imaging [6,7,8,9]. These artifacts negatively affect assessment of Hounsfield Units (HU) which are a radiological measure of tissue density [10]. For radiologists and radio-oncologists this impedes radiological follow-up care as well as radiation therapy planning, since calculation of dose distribution is highly dependent on tissue density assessed based on HU values [11,12,13]. Software algorithms to reduce metal artifacts (MAR) have not been sufficient to fully counteract this effect [14]. Recently, radiolucent polymer mandible reconstruction plates consisting of polyetheretherketone (PEEK), polyetherketoneketone (PEKK), polyphenylsulfone (PPSU) and polyethylene (PE) were found to significantly reduce streak and blooming artifacts in CT images compared to titanium [15]. In an ensuing investigation, it was shown that PEEK, fiber enforced PEEK (f-PEEK) and PEKK provide sufficient fatigue strength to withstand chewing cycles over the cause of at least one year after segmental mandibulectomy [16]. While these are promising results for mandible reconstruction, there are almost no studies analyzing the radiological effects of polymer plates for midfacial reconstruction. The polyaromatic thermoplastic PEEK is ubiquitously used in cranioplasty and craniofacial reconstructive surgery as a light-weight alloplastic onlay graft [17]. PEEK shows good mechanical properties and biocompatibility [18]. Besides its advantageous effect on CT image quality, carbon fiber-reinforced PEEK was shown to reduce dose perturbation in radiation therapy of spinal tumors to less than 5%, compared to more than 30% dose perturbation for titanium [19]. Silicon nitride ceramic-based miniplates for midfacial reconstruction have been shown to cause no artifacts in CT and magnetic resonance (MR) imaging [20]. However, another study found zirconium oxide ceramics to produce strong artifacts in CT images [15]. Therefore, the radiolucent properties of polymers might be more beneficial compared to ceramics. Many different polymer compositions of PEEK, PEKK and PPSU exist for potential medical application. PEEK and PEKK provide fatigue resistance, high yield strength, durability and are lightweight [21]. However, there are no relevant scientific data on many new polymers for patient-specific osteosynthesis in oncologic or traumatic midfacial reconstruction.

Therefore, this study analyzed 12 alloplastic onlay grafts consisting of different polymer compositions for midfacial reconstruction in comparison to a titanium mesh. The aim was to evaluate artifact occurrence and CT image quality compared to a conventional titanium mesh. Additionally, the effect of polymers on radiation dose calculation was analyzed.

## 2. Materials and Methods

### 2.1. Ethical Approval

The Ethics Commission of Heinrich Heine University Düsseldorf gave ethical approval for this experimental study and appointed the trial registration number 2020-993.

### 2.2. Human Cadaveric Specimen

A fresh-frozen (−18 °Celsius [°C]) edentulous human cadaveric skull was obtained from the Institute of Anatomy I of Heinrich Heine University Düsseldorf. Written informed consent for use of the body in experimental medical studies was obtained from the body donor as a standard practice. The skull had no dental or other cranial metallic implants to allow for sole artifact assessment of the inserted implants. For preoperative CT image acquisition and subsequent surgical implantation of titanium and polymer osteosynthesis materials, the human skull specimen was thawed.

### 2.3. Virtual Planning and Manufacturing of Patient-Specific Implants

Digital Imaging and Communications in Medicine (DICOM) dataset files of 1 mm, thin-sliced CT-scans of the skull were generated. These data were used for computer-aided design and manufacturing (CAD/CAM) of all polymer implants.

**Figure 1:**
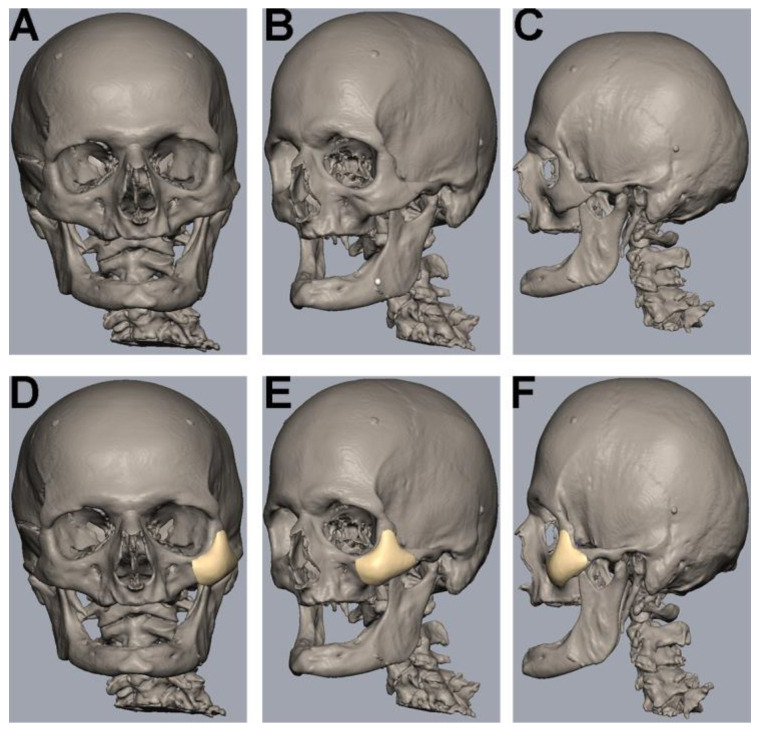
Segmentation, 3D-reconstruction and virtual planning of the zygomatic implant design. (**A**–**C**) Uploading of DICOM data, virtual segmentation and 3D-reconstruction were conducted using the software Individual Patient Solution, IPS Gate^®^ (KLS Martin Group^®^, Tuttlingen, Germany). Conversion of the 3D virtual model to stereolithography (STL) image files was carried out using Mimics 21.0© (Materialise NV, Leuven, Belgium). (**D**–**F**) Defining the zygomatic implant on the left side using a medical modeling software (Geomagic© Freeform Plus© from 3D Systems©, Rock Hill, SC, USA).

The uploading of DICOM data, virtual segmentation and 3D-reconstruction were conducted using the software Individual Patient Solution, IPS Gate^®^ (KLS Martin Group^®^, Tuttlingen, Germany) (Figure 1A–C). The conversion of the 3D virtual model to stereolithography (STL) image files was carried out using Mimics 21.0© (Materialise NV, Leuven, Belgium). Webinar-based (Microsoft© Teams, Redmond, WA, USA) virtual surgery defined the region for the different zygomatic polymer implants on the left side of the skull (Figure 1D–F). The dimensions of all polymer implants were defined using Geomagic© Freeform Plus© (3D Systems©, Rock Hill, SC, USA). The titanium mesh was manually adapted to the zygomatic defect region intraoperatively. All polymer implants were manufactured using additive Fused Filament Fabrication (FFF).

### 2.4. Preparation

Preparation of the zygomatic bone was conducted via a maxillary vestibular approach on the left side only. A horizontal incision from the first incisor to the first molar 5 mm superior to the mucogingival junction was made using a No. 15 blade. Subperiosteal elevators were used to expose the zygomatic bone for an adequate overview. The Osteotomy of the left zygomatic bone was conducted with rotating burrs, without the use of resection guides. The infraorbital nerve was preserved. The defect was then bridged by the different implants as virtually planned preoperatively.

### 2.5. Plates and Screws

**Figure 2:**
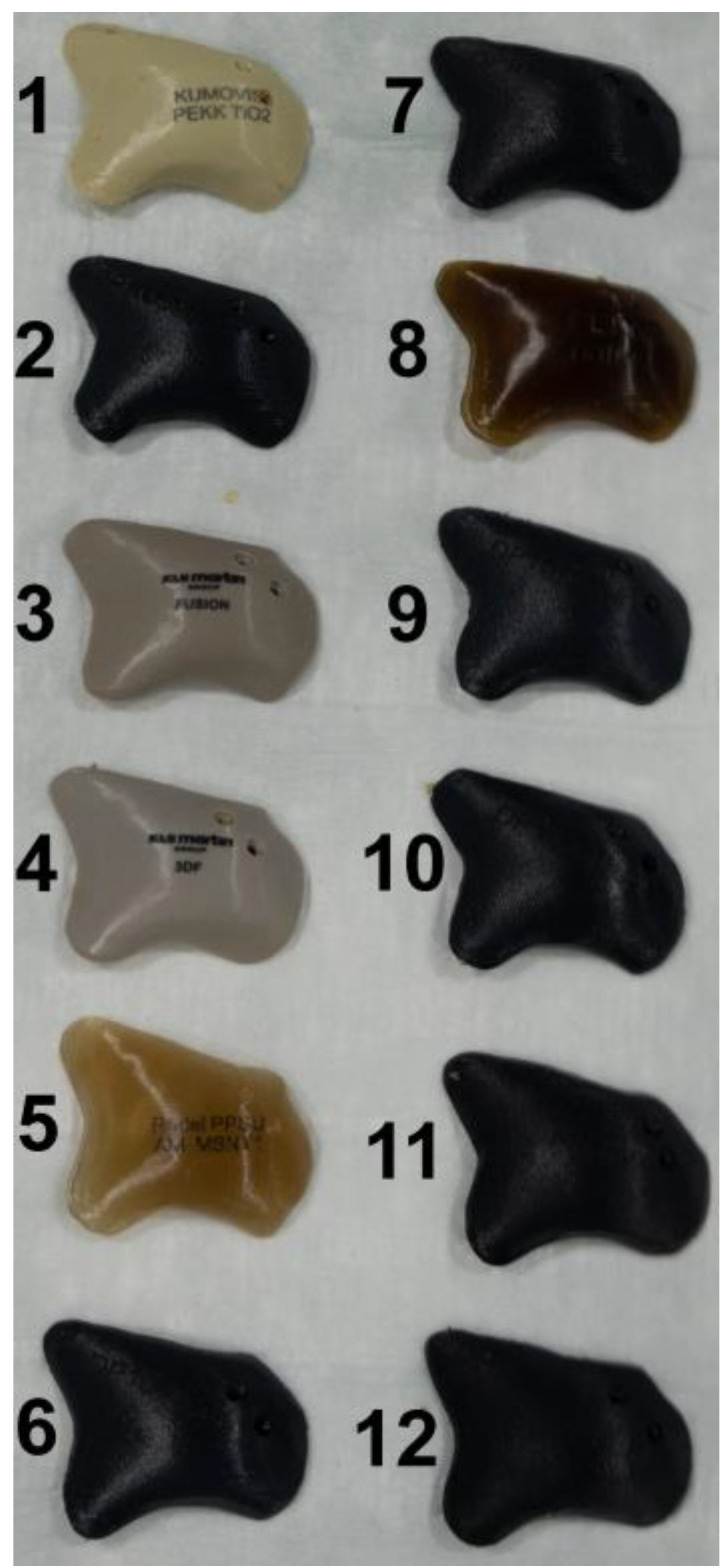
Display of the 12 different zygomatic polymer implants. Numbered: (1) KU PEKK (KUMOVIS GmbH, Munich, Germany); (2) TE PEEK (Ensinger GmbH, Nufringen, Germany); (3) VK iC4800 (EVONIK Industries AG, Essen, Germany); (4) VVK i4 (EVONIK Industries AG, Essen, Germany); (5) Radel PPSU (Solvay GmbH, Hanover, Germany); (6) VK A1 (EVONIK Industries AG, Essen, Germany); (7) VK A2 (EVONIK Industries AG, Essen, Germany); (8) PEKK nature (PEKK Filament, KUMOVIS GmbH, Munich, Germany); (9) VK A3 (EVONIK Industries AG, Essen, Germany); (10) VK B1 (EVONIK Industries AG, Essen, Germany); (11) VK B2 (EVONIK Industries AG, Essen, Germany); (12) VK B3 (EVONIK Industries AG, Essen, Germany).

**Figure 3:**
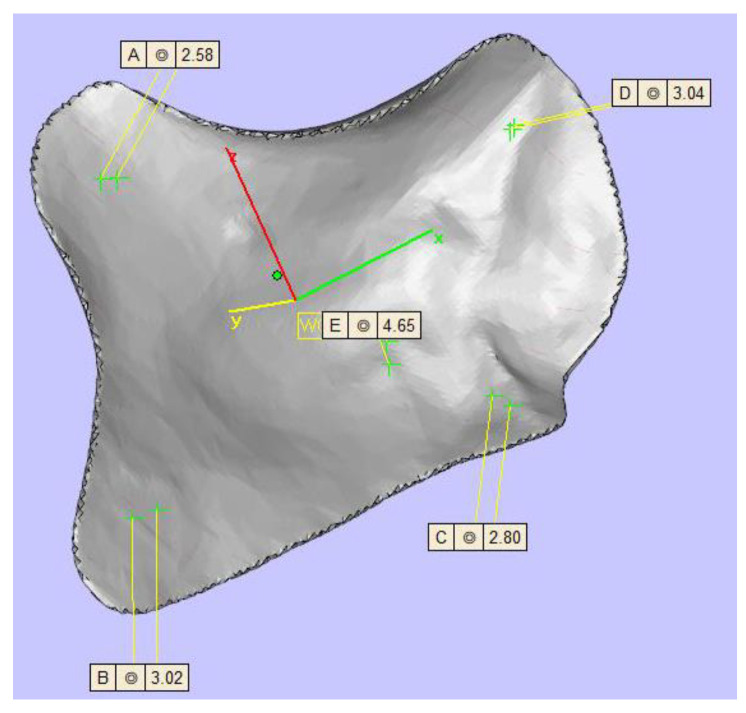
Display of the zygomatic implant design with five different thicknesses (top left 2.58 mm, top right 3.04 mm, center 4.65 mm, left bottom 3.02 mm, right bottom 2.80 mm).

TM (KLS Martin Group^®^, Tuttlingen, Germany), VK iC4800 (EVONIK Industries AG, Essen, Germany), KU PEKK (KUMOVIS GmbH, Munich, Germany), VK i4 (EVONIK Industries AG, Essen, Germany), Radel PPSU (Solvay GmbH, Hannover, Germany), PEKK nature (PEKK Filament, KUMOVIS GmbH, Munich, Germany), TE PEEK (Ensinger GmbH, Nufringen, Germany), VK A1 (EVONIK Industries AG, Essen, Germany), VK A2 (EVONIK Industries AG, Essen, Germany), VK A3 (EVONIK Industries AG, Essen, Germany), VK B1 (EVONIK Industries AG, Essen, Germany), VK B2 (EVONIK Industries AG, Essen, Germany) and VK B3 (EVONIK Industries AG, Essen, Germany) implants were used in this trial (Figure 2). Table 1 provides a list of all implants. For fixation, all implants were fixated with one titanium MAXDrive^®^ screw with a diameter of 1.5 × 8 mm (KLS Martin Group^®^, Tuttlingen, Germany). Screw holes were prepared with a 1.1 × 8 mm irrigated drill. The thickness of all polymer implants was 4.65 mm (Figure 3), and the titanium mesh was 0.45 mm thick.

### 2.6. Computer Tomography (CT) Image Acquisition

CT imaging (Brilliance CT Big Bore^®^, Philips Healthcare, Amsterdam, The Netherlands) of the skull was conducted pre- and postoperatively. Accurate skull positioning in CT was guaranteed by the use of a 1.6 mm thick immobilization mask (MR-03 softfix miniperforation, UNGER Medizintechnik, Mülheim-Kärlich, Germany). CT parameters were set to tube voltage (120 kV), axial scan mode (106 slices with 1 mm slice thickness), detector width (collimation) of 24 mm (16 × 1.5), image resolution 512 × 512 pixels and 0.5 s/circle rotation time. Image reconstruction used a 12-bit CHU scale. All images were reconstructed both with and without the MAR algorithm (Philips Healthcare, Amsterdam, The Netherlands). Since international guidelines only suggest clinical CT slice thicknesses of 2–3 mm, the 1 mm slice thickness of the presented study offers a more sophisticated analysis of CT artifacts [22,23].

### 2.7. Image Analysis

**Figure 4:**
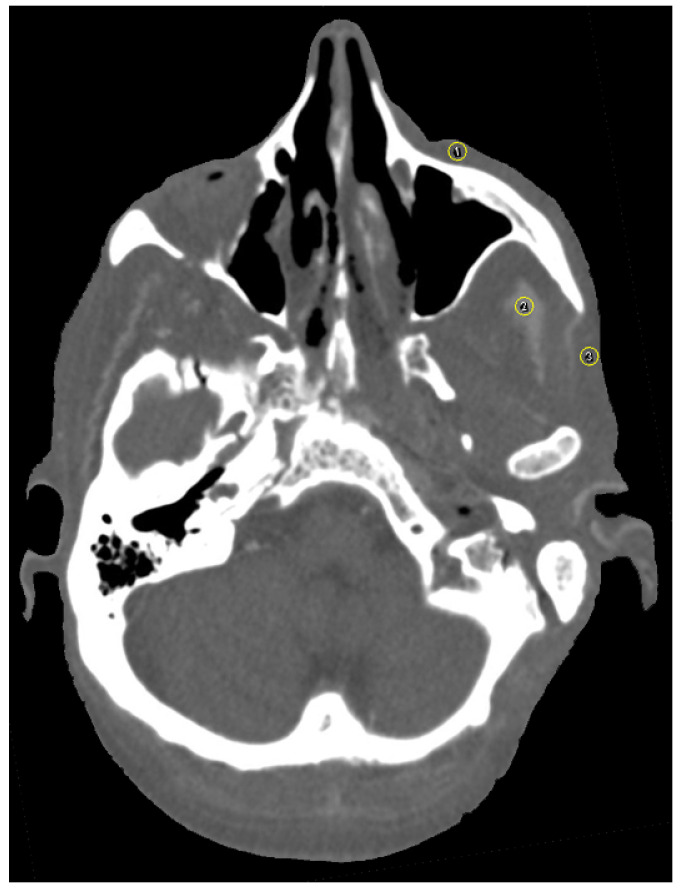
Axial view of selection of three circular regions of interest (ROI) in close proximity to the zygomatic implant using the ROI manager in ImageJ [(1) the zygomatic soft tissue, (2) the temporal muscle and (3) the parotid gland]. Plugins used were CT Window Level and SPICE-CT Package for Computed Tomography QC (Loveland, J.; 2011). HU window was set to soft tissue. Mean HU values were measured for all ROI to determine artifact occurrence.

The software ImageJ (ImageJ 1.48, Wayne Rasband, National Institute of Health, Bethesda, MD, USA) was used for image analysis. As suggested by the software, additional plugins used were CT Window Level and SPICE-CT Package for Computed Tomography QC (Loveland, J.; 2011). All imported DICOM images were analyzed in axial orientation and a specific soft tissue HU window. Streak artifacts occur due to beam-hardening and photon starvation [24] and change HU values. Since beam-hardening artifacts are caused by metallic objects, such as titanium plates, the focus of this study was particularly laid upon streak and blooming artifacts. Patient-based or hardware-based artifacts were not assessed in the design of this study. For artifact assessment, three circular regions of interest (ROI) were selected per image and positioned over (1) the zygomatic soft tissue, (2) the temporal muscle and (3) the parotid gland using the ROI manager (Figure 4). For evaluation of artifact occurrence, mean HU values were measured for all ROI. An image without implants served as a reference for soft tissue HU. Partial volume and beam hardening effects cause blooming artifacts, which are also dependent on the attenuation of the implant [15,25,26]. Tan et al. (2016) and Kasparek et al. (2019) suggested that the best option for the assessment of booming artifacts is the comparison of the actual implant size to the CT measured size [26,27]. This method has been validated in other studies [15]. The virtual growth of all implants in CT images was analyzed by three different radio-oncologists using PACS (IDS7, Sectra AB, Linköping, Sweden). Image quality was further assessed on a five-point Likert scale (1: very good; 2: good; 3: intermediate; 4: poor; 5: very poor).

### 2.8. Statistics

Statistical evaluation was conducted using IBM© SPSS© Statistics for Mac (Version 27; IBM, Armonk, NY, United States). Normality testing was performed using the Shapiro–Wilk test. Data are described as means and standard deviation (SD). Multi-factorial analysis of variance (ANOVA) followed by Bonferroni’s post hoc analysis was used for comparison between groups. A p value < 0.05 was considered statistically significant. The implant material was the primary predictor variable. An image without implant served as control. The prospective HU value was the outcome variable. The median of the Likert scale was used for the analysis of image quality and comparison between implants. The intraclass correlation coefficient (ICC) was used to calculate inter-rater and intra-rater agreement. ICC values were interpreted according to Cicchetti et al. (1994) and Koo et al. (2016) [15,28,29].

## 3. Results

### 3.1. Streak Artifacts

#### 3.1.1. Implant Material

TM (173.7 HU; SD ± 5.1) caused significantly more streak artifacts (measured as mean HU value increase) in CT images compared to control (52.3 HU; SD ± 3.5; *p* < 0.001), KU PEKK (80.7 HU; SD ± 7.4; *p* < 0.001), VK i4 (74.7 HU; SD ± 5.5; *p* < 0.001), Radel PPSU (75.7 HU; SD ± 5.0; *p* < 0.001), PEKK nature (77.0 HU; SD ± 5.3; *p* < 0.001), TE PEEK (74.7 HU; SD ± 6.0; *p* < 0.001), VK A1 (63.3 HU; SD ± 5.9; *p* < 0.001), VK A2 (80.3 HU; SD ± 5.0; *p* < 0.001), VK A3 (68.0 HU; SD ± 7.5; *p* < 0.001), VK B1 (64.7 HU; SD ± 6.7; *p* < 0.001), VK B2 (62.3 HU; SD ± 7.6; *p* < 0.001) and VK B3 (66.0 HU; SD ± 9.5; *p* < 0.001). No statistical difference in streak artifacts between TM (173.7 HU; SD ± 5.1) and VK iC4800 (155.3 HU; SD ± 5.9; *p* < 0.134) was found. There were also no statistical differences between control (52.3 HU; SD ± 3.5) and KU PEKK (80.7 HU; SD ± 7.4; *p* < 0.084), VK i4 (74.7 HU; SD ± 5.5; *p* < 0.123), Radel PPSU (75.7 HU; SD ± 5.0; *p* < 0.125), PEKK nature (77.0 HU; SD ± 5.3; *p* < 0.110), TE PEEK (74.7 HU; SD ± 6.0; *p* < 0.125), VK A1 (63.3 HU; SD ± 5.9; *p* < 0.213), VK A2 (80.3 HU; SD ± 5.0; *p* < 0.085), VK A3 (68.0 HU; SD ± 7.5; *p* < 0.151), VK B1 (64.7 HU; SD ± 6.7; *p* < 0.164), VK B2 (62.3 HU; SD ± 7.6; *p* < 0.243) and VK B3 (66.0 HU; SD ± 9.5; *p* < 0.155).

**Figure 5:**
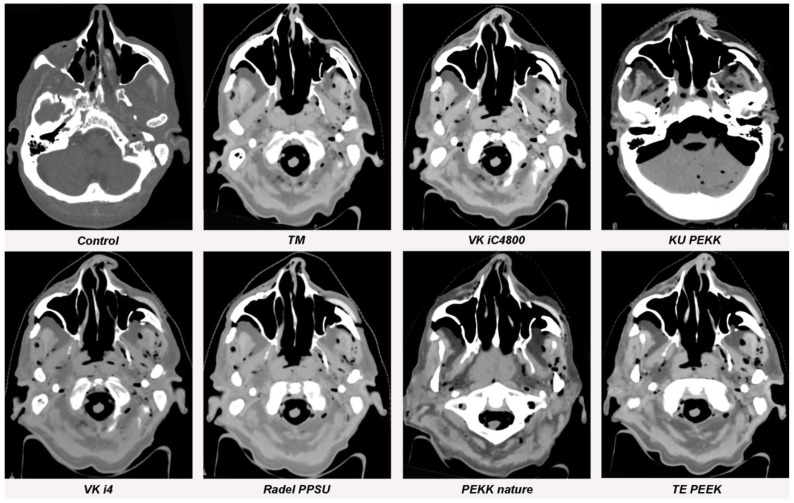
Streak artifacts of TM, VK iC4800, KU PEKK, VK i4, Radel PPSU, PEKK nature and TE PEEK in CT images in axial orientation versus control. Artifacts of each implant were measured at three different anatomical landmarks: (1) the zygomatic soft tissue, (2) the temporal muscle and (3) the parotid gland.

**Figure 6:**
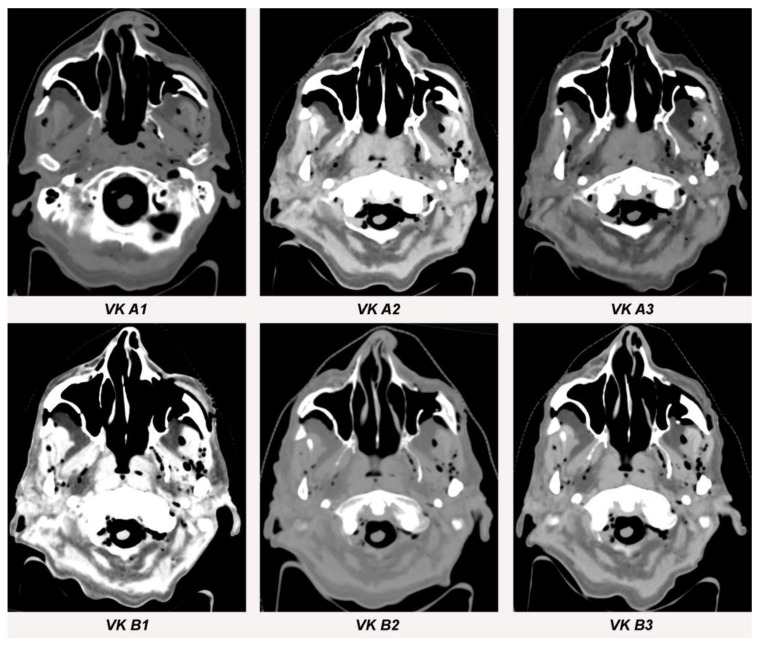
Streak artifacts VK A1, VK A2, VK A3, VK B1, VK B2 and VK B3 in CT images in axial orientation versus control. Artifacts of each implant were measured at three different anatomical landmarks: (1) the zygomatic soft tissue, (2) the temporal muscle and (3) the parotid gland.

**Figure 7:**
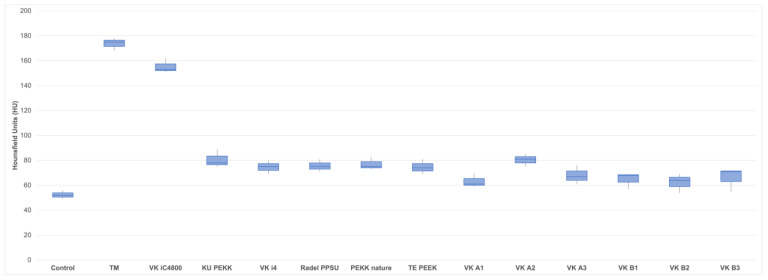
Boxplots of mean Hounsfield Unit (HU) values for all 12 polymer implants (VK iC4800; KU PEKK; VK i4; Radel PPSU; PEKK nature; TE PEEK; VK A1; VK A2; VK A3; VK B1; VK B2; VK B3), TM and control. Mean values are provided in Table 2.

Figure 5, Figure 6 and Figure 7 and Table 2 provide a graphical display of differences in streak artifacts for all implant materials.

#### 3.1.2. Metallic Artifact Reduction Algorithm

CT sequences with and without activation of the metallic artifact reduction algorithm showed no significant differences in streak artifact formation for all implants (TM [*p* = 0.765]; VK iC4800 [*p* = 0.654], KU PEKK [*p* = 0.713]; VK i4 [*p* = 0.574]; Radel PPSU [*p* = 0.798]; PEKK nature [*p* = 0.591]; TE PEEK [*p* = 0.913]; VK A1 [*p* = 0.853]; VK A2 [*p* = 0.576]; VK A3 [*p* = 0.672]; VK B1 [*p* = 0.742]; VK B2 [*p* = 0.611] and VK B3 [*p* = 0.654]).

### 3.2. Blooming Artifacts

#### 3.2.1. Implant Material

No significant blooming artifacts occurred for TM (*p* = 0.987), VK iC4800 (*p* = 0.956), KU PEKK (*p* = 0.956), VK i4 (*p* = 0.987), Radel PPSU (*p* = 0.987), PEKK nature (*p* = 0.932), TE PEEK (*p* = 0.987), VK A1 (*p* = 0.956), VK A2 (*p* = 0.987), VK A3 (*p* = 0.932), VK B1 (*p* = 0.956), VK B2 (*p* = 0.932) and VK B3 (*p* = 0.987). Mean values, standard deviation and virtual growth are displayed in Table 3.

#### 3.2.2. Metallic Artifact Reduction Algorithm

CT sequences with and without activation of the metallic artifact reduction algorithm showed no significant differences in blooming artifact formation for all implants (TM [*p* = 0.654]; VK iC4800 [*p* = 0.923], KU PEKK [*p* = 0.764]; VK i4 [*p* = 0.649]; Radel PPSU [*p* = 0.945]; PEKK nature [*p* = 0.866]; TE PEEK [*p* = 0.791]; VK A1 [*p* = 0.548]; VK A2 [*p* = 0.689]; VK A3 [*p* = 0.779]; VK B1 [*p* = 0.914]; VK B2 [*p* = 0.659] and VK B3 [*p* = 0.760]).

### 3.3. Image Quality

Titanium as well as the polymer implants all showed very good and good image quality. Median image quality for TM, VK iC4800, KU PEKK, Radel PPSU, PEKK nature, VK A1, VK A2, VK A3 and VK B1 was assessed to be good. VK i4, TE PEEK, VK B2 and VK B3 showed very good image quality. Median values for image quality for each material are displayed in Table 3.

## 4. Discussion

Metallic artifacts in CT imaging still pose a major challenge for radiological diagnostics and radiation therapy planning in craniofacial surgery as they reduce CT image quality [30]. Accurate assessment of Hounsfield Units (HU) adjacent to metallic implants is impossible. Hence, the design and manufacture of radiolucent osteosynthesis materials for craniofacial reconstruction have become a prime focus of contemporary scientific research. The results of this study show significantly fewer streak artifacts when polymer instead of titanium implants are used for midfacial reconstruction. The use of a metallic artifact reduction algorithm (MAR) did not significantly influence artifact occurrence in the presented study. Comparable results were already found for PEEK, PEKK, PPSU as well as polyethylene polymer plates in reconstruction after segmental mandibulectomies [15]. While mandible reconstruction streaks and blooming artifacts were reduced likewise by polymer plates [15], blooming artifacts were not significantly reduced by polymer implants in midfacial reconstruction in the presented study. This can possibly be explained by the smaller diameter of titanium meshes used for midfacial reconstruction compared to mandibular titanium reconstruction plates. More than 20 years ago, Ducic (1997) described the use of a titanium mesh in combination with hydroxyapatite cement for midfacial reconstruction [31]. Yet, the results of the presented study found no significant difference in CT artifacts between titanium meshes and VESTAKEEP iC4800 3DF, a polymer that includes hydroxyapatite. Hence, it can be hypothesized that titanium as well as hydroxyapatite are not ideal materials for midfacial reconstruction after tumor reconstructive surgery. Wei et al. (2017) used expanded polytetrafluoroethylene implants to reconstruct maxillonasal dysplasia with satisfactory clinical results [32]. Recently, there have been many trials which have analyzed the use of resorbable polymer plates in midfacial fractures. Schaller et al. (2018) showed that resorbable polylactide-co-glycolide (PLGA) and magnesium plates offer promising results in midfacial fracture healing [33]. Furthermore, the resorbable properties of PLGA and magnesium plates make plate removal superfluous. The PEEK, PEKK and PPSU polymer implants used in the presented study are non-resorbable and mechanically stable which is beneficial after midfacial reconstruction of boney defects, especially after tumor surgery since defect augmentation is intended to be permanent. Furthermore, the individualized design of the implants improves fitting accuracy [34]. Zhang et al., (2022) used patient-specific PEEK implants for paranasal augmentation of midfacial defects with favorable clinical esthetic outcomes [35]. Park at al. (2016) used polyethylene for paranasal augmentation with good clinical outcome [36].

Postoperative irradiation with or without concomitant chemotherapy is often delivered to patients who undergo surgical tumor resection and successive reconstruction of facial bone structures [37]. Malignant bone invasion is a high-risk factor for local recurrence in most of the different tumor types [38]. Any implanted high-density material may cause CT artifacts, leading to inaccuracy of the radiation dose distribution [11]. A time consuming and imprecise manual water density override is often used to compensate metallic artifact effects [39]. Daily adaptation of the radiation plan with the help of MR-linacs [40] and cone beam CT-based high accuracy systems [41] can improve precise radiation therapy. However, an algorithm providing full automatization of organ segmentation and dose planning is required, which is impaired by metallic artifacts [42]. The well-functioning hardware-based artifact reduction of dual energy CTs will unlikely be implemented in cone beam CT-based radiation therapy systems. Particle therapy is emerging due to its favorable dose delivery outside the target volume. Unfortunately, metallic implants also frequently cause considerable error in proton dosimetry [43]. Most of these challenges could possibly be solved by using polymer implants in patients needing adjuvant radiation therapy. This study showed that oral tissues adjacent to polymer implants in the midface can better be predicted with the correct HU in CT imaging. The presented study used 12 different polymer materials for midfacial augmentation which is a satisfactory amount to conduct a differentiated analysis. However, the limitations of the presented study are the use of a cadaver specimen instead of an actual patient to simulate metallic artifact occurrence. Future trials should demonstrate radiation therapy planning protocols for patients with polymer implants to evaluate the actual effect of these implants on radiation therapy.

## 5. Conclusions

Personalized polymer implants for midfacial reconstruction significantly reduce metallic artifacts in CT imaging which improves image quality. Hence, postoperative radiation therapy planning as well as radiological tumor aftercare around the implants can be facilitated. Detailed radiation therapy planning protocols with polymer implants need to be established in the future to assess the clinical effect.

## Figures and Tables

**Table 1:** List of all implant material names, abbreviations and manufacturers.

*Implant Material*	*Abbreviation*	*Manufacturer*
Titanium Mesh	TM	KLS Martin Group^®^, Tuttlingen, Germany
VESTAKEEP iC4800 3DF	VK iC4800	EVONIK Industries AG, Essen, Germany
KUMOVIS PEKK WHITE	KU PEKK	KUMOVIS GmbH, Munich, Germany
VESTAKEEP i4 3DF	VK i4	EVONIK Industries AG, Essen, Germany
Radel PPSU AM MSNT1	Radel PPSU	Solvay GmbH, Hannover, Germany
PEKK nature	PEKK nature	KUMOVIS GmbH, Munich, Germany
TECAFIL PEEK VX CF	TE PEEK	Ensinger GmbH, Nufringen, Germany
VESTAKEEP CF Filament-Fiber A1	VK A1	EVONIK Industries AG, Essen, Germany
VESTAKEEP CF Filament-Fiber A2	VK A2	EVONIK Industries AG, Essen, Germany
VESTAKEEP CF Filament-Fiber A3	VK A3	EVONIK Industries AG, Essen, Germany
VESTAKEEP CF Filament-Fiber B1	VK B1	EVONIK Industries AG, Essen, Germany
VESTAKEEP CF Filament-Fiber B2	VK B2	EVONIK Industries AG, Essen, Germany
VESTAKEEP CF Filament-Fiber B3	VK B3	EVONIK Industries AG, Essen, Germany

**Table 2:** Display of implant materials, mean standard deviation of Hounsfield Units (HU) and their difference to control as well as respective *p*-values.

*Implant Material*	*Abbreviation*	*Mean Standard Deviation (HU)*	*Difference* ^a^	*p-Value* ^a^
Titanium Mesh	TM	173.7 (±5.1)	121.3 (±5.4)	<0.001 *
VESTAKEEP iC4800 3DF	VK iC4800	155.3 (±5.9)	103.0 (±4.2)	0.007 *
KUMOVIS PEKK WHITE	KU PEKK	80.7 (±7.4)	28.3 (±8.5)	0.084
VESTAKEEP i4 3DF	VK i4	74.7 (±5.5)	22.3 (±6.2)	0.123
Radel PPSU AM MSNT1	Radel PPSU	75.7 (±5.0)	23.3 (±7.4)	0.125
PEKK nature	PEKK nature	77.0 (±5.3)	24.7 (±3.3)	0.110
TECAFIL PEEK VX CF	TE PEEK	74.7 (±6.0)	22.3 (±4.3)	0.125
VESTAKEEP CF Filament-Fiber A1	VK A1	63.3 (±5.9)	11.0 (±2.4)	0.213
VESTAKEEP CF Filament-Fiber A2	VK A2	80.3 (±5.0)	28.0 (±6.1)	0.085
VESTAKEEP CF Filament-Fiber A3	VK A3	68.0 (±7.5)	15.7 (±3.3)	0.151
VESTAKEEP CF Filament-Fiber B1	VK B1	64.7 (±6.7)	12.3 (±3.9)	0.164
VESTAKEEP CF Filament-Fiber B2	VK B2	62.3 (±7.6)	10.0 (±2.8)	0.243
VESTAKEEP CF Filament-Fiber B3	VK B3	66.0 (±9.5)	13.7 (±3.1)	0.155

^a^ compared to control; * significance level 0.05.

**Table 3:** Display of implant materials, comparison of real implant diameter (mm) and CT diameter (mm) as virtual growth (mm) with respective *p*-values and image quality.

*Implant Material*	*Abbreviation*	*Real Diameter (mm)*	*CT Diameter (mm)*	*Virtual Growth (mm)*	*p Value*	*Image Quality* ^a^
Titanium Mesh	TM	0.45	0.5 (±0.5)	0.05 (±0.5)	0.987	2
VESTAKEEP iC4800 3DF	VK iC4800	4.65	4.8 (±0.2)	0.15 (±0.2)	0.956	2
KUMOVIS PEKK WHITE	KU PEKK	4.65	4.8 (±0.3)	0.15 (±0.3)	0.956	2
VESTAKEEP i4 3DF	VK i4	4.65	4.7 (±0.1)	0.05 (±0.1)	0.987	1
Radel PPSU AM MSNT1	Radel PPSU	4.65	4.7 (±0.1)	0.05 (±0.1)	0.987	2
PEKK nature	PEKK nature	4.65	4,9 (±0.2)	0.25 (±0.2)	0.932	2
TECAFIL PEEK VX CF	TE PEEK	4.65	4.7 (±0.1)	0.05 (±0.1)	0.987	1
VESTAKEEP CF Filament-Fiber A1	VK A1	4.65	4.8 (±0.1)	0.15 (±0.1)	0.956	2
VESTAKEEP CF Filament-Fiber A2	VK A2	4.65	4.7 (±0.2)	0.05 (±0.2)	0.987	2
VESTAKEEP CF Filament-Fiber A3	VK A3	4.65	4.9 (±0.4)	0.25 (±0.4)	0.932	2
VESTAKEEP CF Filament-Fiber B1	VK B1	4.65	4.8 (±0.2)	0.15 (±0.2)	0.956	2
VESTAKEEP CF Filament-Fiber B2	VK B2	4.65	4.9 (±0.1)	0.25 (±0.1)	0.932	1
VESTAKEEP CF Filament-Fiber B3	VK B3	4.65	4.7 (±0.1)	0.05 (±0.1)	0.987	1

^a^ Five-point Likert scale with median ranking (1–5; 1 = very good; 2 = good; 3 = intermediate; 4 = bad; 5 = very bad).

## Data Availability

The datasets used and analyzed during the current study are available from the corresponding author upon reasonable request.
